# Neurovascular therapeutic potential of neuromodulation in Alzheimer’s disease

**DOI:** 10.4103/NRR.NRR-D-25-00958

**Published:** 2025-12-30

**Authors:** Maria Luisa De Paolis, Claudio Zaccone, Marcello D’Amelio

**Affiliations:** Department of Medicine and Surgery, Università Campus Bio-Medico di Roma, Rome, Italy; Department of Experimental Neurosciences, IRCCS Santa Lucia Foundation, Rome, Italy

Alzheimer’s disease (AD) has been traditionally viewed as a purely neuronal pathology, marked by synaptic loss, amyloid-β (Aβ) accumulation, tau tangles, neuroinflammation, and metabolic imbalance. Over the past two decades, cerebrovascular dysfunction has emerged as both a co-initiator and an amplifier of AD pathology. Structural and functional perturbations within the neurovascular unit (NVU) — a dynamic interface of vascular, glial, and neuronal cells that coordinates cerebral blood flow (CBF), maintains blood–brain barrier (BBB) integrity, and regulates neuronal and synaptic activity — can precede overt cognitive symptoms and contribute to the collapse of cerebral homeostasis (Zlokovic et al., 2011; Luo et al., 2022). Accordingly, neurodegeneration and cerebrovasculopathy evolve in parallel and may reinforce each other, acting either independently and/or in synergy with Aβ accumulation. This is particularly evident in regions such as the brainstem, where the vascular architecture is uniquely vulnerable: paramedian perforating vessels arise directly from major arterial trunks, lack collateralization, and possess thin walls prone to hypertension-induced damage. These features expose these regions to elevated shear stress, spontaneous microbleeds and ischemic injury, contributing to their selective vulnerability in aging and dementia. In AD, such vascular fragility may underlie the early degeneration of the isodendritic core nuclei and the dysfunction of their projection fields (Zaccone et al., 2025).

Amid this complex interplay, non-invasive brain stimulation (NIBS) techniques, such as transcranial electrical stimulation (tES) and repetitive transcranial magnetic stimulation (rTMS), emerge as promising neuromodulatory tools. Traditionally employed to modulate neuronal excitability and cognitive performances, NIBS also shows the ability to modulate non-neuronal targets, including components of the NVU. Indeed, preclinical and clinical studies demonstrate that specific stimulation paradigms can restore CBF, upregulate angiogenic factors, and preserve BBB integrity, highlighting the vascular system as a potential mediator of therapeutic efficacy (Iyer et al., 2018; Petrovskaya et al., 2023).

In this perspective, we advance a reconceptualization of AD treatments by integrating vascular mechanisms into the therapeutic rationale for brain stimulation. We synthesize emerging evidence of NIBS-induced vascular remodeling, and advocate for future stage-specific, NVU-targeted strategies that harness the full neurovascular therapeutic potential of neuromodulation in AD.

**Dynamic shifts in angiogenesis across the Alzheimer’s disease timeline – the self-amplifying loop:** Vascular remodeling in AD reflects a stage-dependent spectrum, transitioning from early capillary rarefaction to late pathological neovascularization.

**Figure 1:**
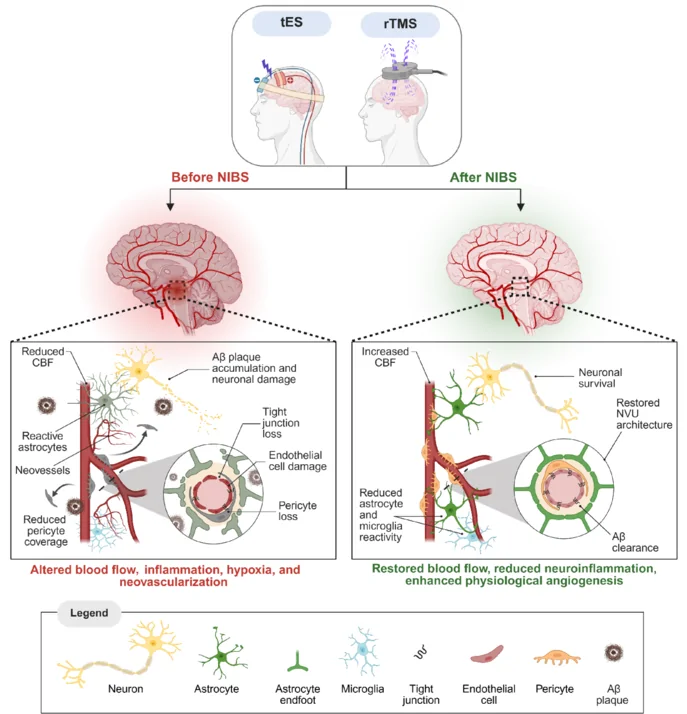
Effects of NIBS on NVU integrity in AD. Among NIBS techniques, tES and rTMS pose future potential in the “vasculostimulation” field of AD therapy. The brainstem — outlined with a dotted square — is marked by a red dot in the pathological pre-NIBS condition, as it represents an early site of neurovascular dysfunction where these effects may converge. Pathological changes in AD, in the absence of NIBS treatment, include a reduced CBF, pericyte loss, and astrocytic disconnection, accompanied by the BBB breakdown and formation of dysfunctional neovessels. These alterations promote Aβ deposition, endothelial damage, neuroinflammation, and neuronal death. Following NIBS intervention, increased CBF and glial modulation contribute to BBB restoration, NVU stabilization, and potentially enhanced physiological angiogenesis, thereby supporting Aβ clearance and neuronal survival. Created with BioRender.com. Aβ: Amyloid-β; AD: Alzheimer’s disease; BBB: blood–brain barrier; CBF: cerebral blood flow; NIBS: non-invasive brain stimulation; NVU: neurovascular unit; rTMS: repetitive transcranial magnetic stimulation; tES: transcranial electrical stimulation.

***Presymptomatic phase and mild cognitive impairment:*** Histological and imaging data reveal a consistent pattern of reduced capillary density and diminished CBF, accompanied by dysregulation of key proangiogenic factors, such as vascular endothelial growth factor and angiopoietin-2. This angiogenic insufficiency coincides with early disruption of tight junctions, which promotes BBB leakage and establishes a feedforward cascade wherein oxidative stress depletes endothelial nitric oxide, perpetuating cerebral hypoperfusion and amplifying oxidative stress. The BBB breakdown permits infiltration of peripheral immune cells and manifests as cerebral microbleeds, especially in the hippocampus and cortex. Concomitantly, structural degradation of the NVU occurs; pericyte loss, endothelial dysfunction, and astrocytic detachment compromise cerebrovascular homeostasis, reducing perfusion capacity (**Figure 1**). The converging vascular and neurodegenerative insult progressively lowers the threshold for cognitive decline, especially in the context of comorbid vascular risk, e.g., hypertension and diabetes (Zlokovic et al., 2011).

***Moderate Alzheimer’s disease:*** This disease stage is characterized by endothelial senescence, reduced pericyte coverage, and heightened BBB permeability, alongside a shift toward pro-inflammatory angiogenesis driven by cytokines and matrix-degrading enzymes, fostering a hypoxic, pro-oxidative environment detrimental to brain metabolism (Zlokovic et al., 2011).

***Advanced Alzheimer’s disease:*** Transcriptomic analyses reveal an upregulation of genes associated with endothelial cell proliferation and neovessel sprouting. The newly formed microvessels are structurally defective as they exhibit aberrant branching patterns and often contribute to a worsened perfusion mismatch, whereby CBF fails to adequately meet the metabolic demands of the surrounding neural tissue (**Figure 1**). This maladaptive neovascularization exacerbates inflammation, aggravates hypoxia, and accelerates neurodegeneration (Zlokovic et al., 2011).

Collectively, these alterations delineate a self-reinforcing pathological loop. Vascular dysfunction impairs nutrient and oxygen delivery to neurons, destabilizes metabolic balance, and hinders Aβ clearance, thereby amplifying its extracellular and vascular accumulation. This sequence aligns with the “two-hit vascular hypothesis” (Zlokovic et al., 2011): cerebrovascular injury initiates neuronal dysfunction (hit one) and facilitates Aβ accumulation (hit two), ultimately driving a feedforward neurodegenerative cascade. In turn, Aβ-induced endothelial toxicity, perivascular Aβ accumulation (determining cerebral amyloid angiopathy), and glial inflammation further degrade the NVU, closing a vicious cycle that progressively broadens neural vulnerability (**Figure 1**).

Interrupting this self-perpetuating cycle requires therapeutic strategies that concurrently foster vascular competence and neuronal resilience. Thus, the clinical approach should be redefined, not as sequential steps but as simultaneous interventions targeting the neurovascular interface.

**Neuromodulation beyond neurons – evidence for neurovascular unit modulation by non-invasive brain stimulation:** NIBS techniques have recently gained increasing traction as promising interventions for AD, with encouraging results on cognitive outcomes. Mechanistically, NIBS modulate neuronal function and synaptic plasticity by altering cortical excitability, regulating neurotransmitter release, and engaging molecular pathways critical for synaptic strengthening and network reorganization (Petrovskaya et al., 2023; De Paolis et al., 2024).

Among the most studied tools, rTMS and tES have shown measurable, albeit variable, clinical efficacy. Specifically, rTMS generates focal electric fields capable of depolarizing cortical neurons. Several clinical studies report rTMS-induced cognitive improvements, with benefits often sustained post-intervention, especially in the early stages of AD (Petrovskaya et al., 2023). Similarly, tES — which encompasses techniques such as transcranial direct current stimulation (tDCS), delivering constant unidirectional and low-intensity currents, and transcranial alternating current stimulation (tACS), applying oscillatory currents to entrain endogenous neuronal rhythms in a frequency-specific manner — has been increasingly recognized as a versatile class of interventions capable of modulating brain activity by applying weak electrical currents across the scalp. By subtly shifting neuronal membrane potentials, tES can influence the excitability of targeted cortical regions and facilitate endogenous neuroplastic mechanisms (Petrovskaya et al., 2023; De Paolis et al., 2024).

However, the growing interest in NIBS neuronal effects should not eclipse other equally relevant, yet less explored, non-neuronal actions. Accumulating evidence suggests that the outcomes of these techniques extend beyond neurons to include vascular components, impacting CBF, BBB integrity, and the activity of cells within the NVU, breaking the vicious circle driving AD pathology (**Figure 1**; Petrovskaya et al., 2023). Despite the growing relevance of these vascular effects, they are often considered ancillary to cognitive endpoints and remain largely underexplored in both research and clinical practice. To date, few studies have specifically and exclusively investigated the vascular mechanisms of NIBS in the context of AD, highlighting a critical gap in our understanding of their full therapeutic potential.

Notably, among the available neuromodulation techniques, focused ultrasound stimulation (FUS) is the most extensively studied in relation to BBB permeability, NVU engagement, and drug delivery in AD, with promising preclinical and early clinical data supporting its biological and cognitive effects (for a review, see Patwardhan et al., 2024). FUS applies mechanical acoustic energy to brain tissue, achieving millimeter-level spatial precision and deep penetration into subcortical targets. FUS, particularly when combined with systemically administered microbubbles, transiently opens the BBB through mechanical effects on endothelial tight junctions, thereby enhancing molecular transport and modulating neurovascular dynamics (Patwardhan et al., 2024). However, the high cost, limited availability, and technical complexity of FUS protocols currently restrict their widespread application. For this reason, non-invasive, low-cost, and easily deployable alternatives, even potentially applicable at home, deserve systematic investigation for their potential to achieve neurovascular benefits in a more accessible and scalable way.

Accordingly, in rodent models, tDCS shows bidirectional influence on CBF in a polarity- and intensity-dependent manner, with anodal stimulation increasing perfusion and cathodal stimulation reducing it. Recent *in vivo* two-photon imaging demonstrates that these effects directly modulate microvessels exhibiting spontaneous vasomotion, with anodal stimulation inducing rapid, reversible vasodilation, increased blood flux, and a dose-dependent rise in cortical microvascular permeability (Gellner et al., 2022). These vascular responses persist beyond stimulation and are accompanied by enhanced perivascular microglial motility, suggesting straight-on engagement of NVU components beyond neuron-driven neurovascular coupling, the bidirectional mechanism aligning local CBF with metabolic demand. Such targeted microvascular actions may also involve astrocyte engagement, whose signaling contributes to the integrated response of all the NVU components. This hypothesis is also supported by a study in the APP/PS1 mouse model of AD, where anodal tDCS elicited a remodeling of the NVU, restoring cerebrovascular architecture. Glial tDCS-driven modulation is similarly pronounced, with reduced microglial and astrocytic activation and relocation of aquaporin-4 in astrocytic endfeet. These changes coincide with reduced cortical and hippocampal Aβ burden and are associated with a shift in APP processing towards the non-amyloidogenic pathway. The upregulation of lipoprotein receptor-related protein 1 at the BBB emerges as a key effector, linking vascular repair to enhanced Aβ clearance (Luo et al., 2022). This is particularly relevant considering that impaired clearance, rather than overproduction, is likely the main driver of Aβ accumulation in sporadic AD (Zlokovic et al., 2011).

We recently demonstrated that prefrontal anodal tDCS activates the ventral tegmental area (De Paolis et al., 2025), a deep brainstem nucleus known to undergo early degeneration in AD (Nobili et al., 2017). In Tg2576 mice, this stimulation re-engages ventral tegmental area neuronal activity and boosts evoked dopamine release in projection areas, reducing neuroinflammation and amyloid pathology. These findings suggest a potential neuroprotective mechanism driven by the reactivation of dopaminergic signaling. Notably, the observed effects may be sustained by both direct neuromodulatory influences and vascular adaptations initiated cortically and conveyed via cortico-mesencephalic pathways, ultimately enhancing perfusion and NVU integrity within the brainstem itself.

Insights from preclinical stroke models, which share with AD key features, such as endothelial dysfunction, neuroinflammation, and perfusion deficits, offer further translational support. Indeed, in a rodent model of subacute stroke, low-frequency rTMS triggers the upregulation of angiopoietin-1 and synaptophysin within the ischemic core, alongside increased phosphorylation of protein kinase B and endothelial nitric oxide synthase in both the infarct and peri-infarct areas. In this experimental paradigm, the transcription of the endothelial angiopoietin-1 receptor is also enhanced, supporting the role of rTMS in the stabilization and maturation of nascent vasculature (Lee et al., 2022).

Further clinical evidence confirms the vascular effects of NIBS. In healthy individuals, intermittent theta burst stimulation, a specific protocol of rTMS, enhances cerebrovascular reactivity to CO₂ without altering baseline CBF, implying a selective modulation of microvascular tone, possibly via autonomic or astrocyte-mediated pathways (Iyer et al., 2018). Additional clinical support for the vascular actions of NIBS comes from systematic analyses of transcranial Doppler studies in post-stroke populations. These investigations consistently reveal that both anodal tDCS and low-frequency rTMS acutely modulate CBF. Depending on stimulation site and polarity, NIBS protocols demonstrate the ability to either increase perfusion in the lesioned hemisphere or rebalance interhemispheric flow asymmetries — effects that are more pronounced in the subacute phase and, in some cases, persist after repeated sessions (Iyer et al., 2018).

Although less explored, tACS also demonstrates cerebrovascular outcomes. In preclinical settings, this technique enhances regional CBF and oxygen saturation, with more substantial and prolonged effects in awake *versus* anesthetized mice (Bragina et al., 2023). In patients with mild-to-moderate AD, 40 Hz tACS over the temporal lobes increases perfusion, as assessed by arterial spin labelling-magnetic resonance imaging, with these changes correlating with episodic memory improvements and gamma power augmentation (Sprugnoli et al., 2021).

Collectively, these findings reinforce a paradigm shift: BBB modulation and vascular plasticity may not be collateral consequences of NIBS, but rather primary therapeutic targets, particularly in early or prodromal stages of AD where vascular decline is prominent yet potentially reversible.

The dual identity of AD as both a neurodegenerative and neurovascular disorder demands therapeutic strategies that target neuronal, vascular, and glial components in concert. NIBS is emerging as a promising approach capable of modulating neural activity while restoring NVU integrity, prompting a shift toward stimulation paradigms optimized for disease stage and vascular status. Although all NIBS modalities converge on neural modulation, they differ substantially in physical mechanisms, spatial resolution, and vascular engagement.

For instance, tDCS exerts polarity-dependent effects on CBF and BBB integrity, with vascular actions demonstrated in preclinical models and early-phase AD trials. tACS has shown preclinical and clinical evidence of perfusion enhancement, while rTMS can promote angiogenic signaling and regulate microvascular tone. FUS enables transient, localized BBB opening, though its use remains largely confined to specialized centers and early-stage trials. Selection of the appropriate modality should therefore be guided not only by the neuronal target but also by the desired neurovascular effect and the clinical context, balancing efficacy with feasibility.

NIBS can influence both pre-existing and inducible molecular pathways within the NVU. For example, tDCS-mediated upregulation of astrocytic aquaporin-4 and endothelial lipoprotein receptor-related protein 1 may couple vascular repair with enhanced Aβ clearance, a potentially valuable mechanism despite the limited therapeutic impact of amyloid reduction in advanced disease. In earlier stages, engaging angiogenic and endothelial pathways, such as vascular endothelial growth factor activation and endothelial nitric oxide synthase-driven nitric oxide production, may improve perfusion, promote physiological angiogenesis, and stabilize the BBB. While direct experimental validation of these processes in AD remains limited, converging evidence implicates additional, yet uncharacterized, molecular mediators capable of coordinating vascular and glial responses throughout disease progression.

Given the dynamic progression of vascular pathology across the AD continuum, NIBS-based interventions may require stage-specific tailoring, targeting distinct neurovascular mechanisms at different points in the disease progression.

***Early/prodromal phases:*** When reduced perfusion and vascular rarefaction prevail, protocols that support endothelial function and enhance physiological angiogenesis may help restore NVU architecture. Thus, restoring vascular integrity and optimizing flow dynamics may delay neurodegeneration and prevent the amplification of downstream amyloid and tau pathology.

***Advanced stages:*** Characterized by maladaptive and leaky angiogenesis, this phase shifts the therapeutic focus toward stabilizing the BBB, limiting inflammation-driven neovascularization, and restoring NVU balance — possibly requiring adjusted stimulation intensities or frequencies to avoid exacerbating vascular leakage due to the potential of NIBS in increasing cerebral perfusion.

Mechanistically, NIBS may influence vascular function through (i) direct modulation of NVU elements and BBB permeability, including polarity-dependent effects on CBF, moving beyond classical neurovascular coupling, and (ii) indirect, circuit-driven pathways, such as activation of subcortical hubs like the ventral tegmental area. The latter effects are more likely with protocols that engage deep targets or modulate top-down control circuits. Notably, the idiosyncrasies of the BBB in the brainstem nuclei should be taken into account, as they may render these regions unusually susceptible, or receptive, to neuromodulatory inputs, positioning dopaminergic circuitry as both mediator and biomarker of vascular function.

Direct and indirect mechanisms may act independently or interactively, with vascular engagement potentially priming the NVU for neuronal modulation, or *vice versa*. Clarifying their temporal and mechanistic interplay will require integrated experimental designs combining cell-specific molecular assays with vascular imaging and functional readouts. Furthermore, because most “vasculostimulation” evidence derives from non-AD contexts, BBB responsiveness must be interrogated across different stimulation parameters, loci, and AD stages. Rigorous biomarker integration — combining arterial spin labelling-magnetic resonance imaging, dynamic-contrast imaging, and plasma indices of endothelial activation and dopaminergic tone — will be pivotal for stratification and real-time monitoring.

Ultimately, multimodal protocols combining NIBS with pharmacological or lifestyle interventions may help reinstate NVU homeostasis, thereby preserving neurovascular coupling throughout the entire AD continuum.

*This work was supported by the American Alzheimer’s Association [AARG-18-566270; AARG-21-851219], by the Italian Ministry of Health [Research Grant: RF-2018-12365527], by Regione Lazio [PO FESR LAZIO 2014/2020, T0002E0001], by the Italian Ministry of Universities and Research [Prot. 2020Z73J5A], and by Fondazione Roma (Rome, Italy) (all to MDA).*
